# Anti-PD-1 antibody decreases tumour-infiltrating regulatory T cells

**DOI:** 10.1186/s12885-019-6499-y

**Published:** 2020-01-08

**Authors:** Kazushige Yoshida, Masanori Okamoto, Jun Sasaki, Chika Kuroda, Haruka Ishida, Katsuya Ueda, Hirokazu Ideta, Takayuki Kamanaka, Atsushi Sobajima, Takashi Takizawa, Manabu Tanaka, Kaoru Aoki, Takeshi Uemura, Hiroyuki Kato, Hisao Haniu, Naoto Saito

**Affiliations:** 1 Department of Orthopaedic Surgery, Shinshu University School of Medicine, Matsumoto, Japan; 2 Institute for Biomedical Sciences, Interdisciplinary Cluster for Cutting Edge Research, Shinshu University, Asahi 3-1-1, Matsumoto, 390-8621 Japan; 3 Department of Orthopedic Surgery, Okaya City Hospital, Okaya, Japan; 4 Physical Therapy Division, School of Health Sciences, Shinshu University School of Medicine, Matsumoto, Japan

**Keywords:** PD-1, Treg, Osteosarcoma, Anti-PD-1 antibody

## Abstract

**Background:**

There are many types of therapies for cancer. In these days, immunotherapies, especially immune checkpoint inhibitors, are focused on. Though many types of immune checkpoint inhibitors are there, the difference of effect and its mechanism are unclear. Some reports suggest the response rate of anti-PD-1 antibody is superior to that of anti-PD-L1 antibody and could potentially produce different mechanisms of action. On the other hand, Treg also express PD-1; however, their relationship remains unclear.

**Methods:**

In this study, we used osteosarcoma cell lines in vitro and osteosarcoma mouse model in vivo. In vitro, we analyzed the effect of IFNγ for expression of PD-L1 on the surface of cell lines by flowcytometry. In vivo, murine osteosarcoma cell line LM8 was subcutaneously transplanted into the dorsum of mice. Mouse anti-PD-1 antibody was intraperitoneally administered. we analysed the effect for survival of anti-PD-1 antibody and proportion of T cells in the tumour by flowcytometry.

**Results:**

We discovered that IFNγ increased PD-L1 expression on the surface of osteosarcoma cell lines. In assessing the relationship between anti-PD-1 antibody and Treg, we discovered the administration of anti-PD-1 antibody suppresses increases in tumour volume and prolongs overall survival time. In the tumour microenvironment, we found that the administration of anti-PD-1 antibody decreased Treg within the tumour and increased tumour-infiltrating lymphocytes.

**Conclusions:**

Here we clarify for the first time an additional mechanism of anti-tumour effect—as exerted by anti-PD-1 antibody decreasing Treg— we anticipate that our findings will lead to the development of new methods for cancer treatment.

## Backgrounds

In the microenvironment of cancer, the role of innate immunity is inhibited in a process known as immune tolerance. One of the mechanisms of immune tolerance is the immune checkpoint mechanism, whereby T cells are suppressed to prevent excessive immune responses. Several types of immune checkpoint molecules are known, namely the cytotoxic T-lymphocyte antigen 4 (CTLA-4) and lymphocyte activation gene 3 (LAG-3), in addition to programmed cell death 1 (PD-1) and its ligand 1 (PD-L1) [1, 2]. PD-1 is expressed on the surface of cytotoxic T cells and transmits suppressive signals to T cells by binding to PD-L1.

Normal cells are believed to express PD-L1 in an inflammatory environment, suppress T cells, and prevent excessive tissue damage from long-term persistence and spread of inflammation [3]. However, in some types of cancers, PD-L1 is reported to be expressed on the surface of cancer cells by means of stimulation from interferon gamma (IFNγ), a proinflammatory cytokine [1, 4–6]. Cancer has been implicated to prevent attacks from the immune system by suppressing T cell activation by binding the PD-L1 that are expressed on cancer cells to the PD-1 on cytotoxic T cells [7]. Therefore, when anti-PD-1 or anti-PD-L1 antibodies are allowed to react to a specified antigen, PD-1 is unable to bind to PD-L1, and an anti-tumour effect is exerted by disabling their immunotolerance [8]. To date, two reports have analysed a study population of over 3000 patients. Although the reported effect of anti-PD-1 and anti-PD-L1 antibodies were equivalent in a 2017 study [9], a 2018 study showed that the response rate of anti-PD-1 was superior to that of anti-PD-L1 antibody [10]. These results indicate that different mechanisms of action may exist as the anti-PD-1 antibody suppresses tumours.

Immune checkpoint molecules also play an important role in Treg that are involved in suppressing the function of cytotoxic T cells. Treg expresses CTLA-4, which is an immunity checkpoint molecule on the cell surface that suppresses the activity of antigen-presenting cell (APC), resulting in the suppression of T cell activation [11]. The anti-tumour effect of anti-CTLA-4 antibody is obtained by the inhibition of CTLA-4 on Treg and thus reversing the suppression of T cell activation [12, 13]. Some reports have noted the expression of PD-1 on the surface of Treg [14–17], and the importance of PD-1 on Treg have been pointed [16, 18]. Although there are few comprehensive studies that describe the relationship between anti-PD-1 antibody and Treg [19], the effect of anti-PD-1 antibody on Treg is not clear. In regards to the therapeutic effect of anti-PD-1 antibody against osteosarcoma, there are only three interim reports on clinical trials [20–22] and one basic research report [23]. Moreover, although PD-L1 is reportedly expressed in osteosarcoma [24], its expression mechanism is unknown.

This study used osteosarcoma as a tumour model to elucidate its relationship to the anti-tumour effect of anti-PD-1 antibody and Treg. Osteosarcoma is reported as a tumour that is susceptible to immunotherapy [25] with greater infiltration of CD8 + cells than other sarcomas [26], and prognosis is considered better when there is more infiltration of CD8 + cells [27]. Because osteosarcoma is a solid tumour, it is suitable for evaluating the relationship between anti-PD-1 antibody and Treg in animal experiments.

In this study, we first evaluated the mechanism of PD-L1 expression in osteosarcoma cells in vitro to establish osteosarcoma as a tumour model. The anti-tumour effect was also confirmed in vivo from the changes in tumour volume and overall survival time of anti-PD-1 antibody administration using a subcutaneously implanted mouse model of osteosarcoma. Furthermore, changes in the tumour microenvironments were evaluated in detail, and the relationship between anti-PD-1 antibody and Treg was examined. From these experiments, we report that anti-PD-1 antibody suppresses tumour-infiltrating Treg and exerts an antitumour effect.

## Methods

### Cell lines

Murrin osteosarcoma cell line (LM8, Riken cell bank, Tokyo, Japan, RBC Cat# RCB1450), human osteosarcoma cell lines HOS (Riken cell bank, RBC Cat# RCB0428, RRID:CVCL_0312) and SaOS-2 (Riken cell bank, RCB Cat# RCB0992, RRID:CVCL_0548) were cultured in α-MEM containing 5% fetal bovine serum (FBS). 143B human osteosarcoma cells (Riken cell bank, RCB Cat# RCB0701, RRID:CVCL_2270) were cultured in Dulbecco’s Modified Eagle Medium (DMEM) containing 5% FBS. All cell cultures were maintained in 5% CO2 at 37 °C. For all cell lines, frozen aliquots of 10^6 cells were stored at a passage below 5 and a fresh aliquot used after 5 consecutive passages. All of cell lines were additionally tested by Riken cell bank and found to be negative for mycoplasma.

### Mice

Mice were housed and maintained at the Committee for Animal Experiments of Shinshu University. Mice were housed with an inverse 12 h day-night cycle with lights on at 9 am in a temperature-controlled room. All mice were allowed free access to water and a maintenance diet. Based on the national regulations and guidelines, all experimental procedures were reviewed by the Committee for Animal Experiments and finally approved by the president of Shinshu University. The animal protocol was approved by the Committee for Animal Experiments of Shinshu University (Approval Number 280112).

Male C3H/HeSlc mice (3 weeks of age, 13.59 ± 1.13(Body weight ± S.D.)) were bought by Japan SLC (Shizuoka, Japan). The established protocol [28] is used for the standardized protocol for euthanasia.

### IFNγ stimulation and PD-L1 expression in osteosarcoma cell lines

Recombinant IFNγ (R & D systems, Minneapolis, MN, USA) species-matched to human osteosarcoma cell lines (HOS, SaOS-2, 143B) and murine osteosarcoma cell line (LM8) was administered and incubated for 24 h (*n* = 3). Cells were detached with trypsin, and surface markers were detected by flow cytometry.

### Administration of 4H2 on osteosarcoma mouse model

Murine osteosarcoma cell line LM8 was subcutaneously transplanted into the dorsum of 4-week old mice (1 × 10^6^ cells per mouse, *n* = 5). Mouse anti-PD-1 antibody (4H2, Ono Pharmaceutical Co., Osaka, Japan) was intraperitoneally administered three times per week at 20 mg/kg of body weight per dose for a total of five doses. 200 μl of PBS was intraperitoneally administered to the control group. Mice were followed up until reaching the humane endpoint or until natural death. Macroscopic tumour size was calculated using the established method [29] three times a week. Body weight, and μCT were observed over time.

### Tumour microenvironment after administration of 4H2

Subcutaneously transplanted mouse models of osteosarcoma were prepared (*n* = 3). To obtain a sufficient amount of tumour at 2 weeks after the initial administration of 4H2, the initial administration was withheld until day 7. 4H2 was intraperitoneally administered three times per week at 20 mg/kg of body weight per dose for a total of five doses. 200 μl of PBS was intraperitoneally administered to the control group. All mice were euthanized at day 21, and the tumour and spleen were excised.

### Long-term administration of 4H2 on osteosarcoma mouse model

Subcutaneously transplanted mouse models of osteosarcoma were prepared (*n* = 5). In the short-term administration group, 4H2 was intraperitoneally administered twice a week at 20 mg/kg per dose for a total of four doses. In the long-term administration group, administration of anti-PD-1 antibody was continued twice a week until the endpoint. For the control group, PBS was administered twice a week until the endpoint. Mice were followed up until reaching our euthanasia criteria or until natural death. Changes in tumour size, body weight, and μCT were observed over time.

### Antibodies, reagents, and flow cytometry

Fluorochrome-conjugated antibodies and reagents used in this research are described in Additional file 1: Table S1. Foxp3 Transcription Factor Staining Buffer Set (eBioscience, San Diego, CA, USA) was used for intracellular staining of Foxp3 and Ki-67. Stained cells were analysed with FACS Canto II flow cytometer or FACS Cellesta flow cytometer (BD Bioscience, Haryana, India). To collect and analyse the data, well established method of multicolor flow cytometry [30] was used. Flow cytometry data were analysed with Kaluza software ver.1.5a (Beckman Coulter, Brea, CA, USA).

### Cell isolation

Mice were euthanized by inhaled isoflurane overdose. The tumour of the dorsal subcutaneous and spleen were excised in a clean environment. The excised spleen was physically triturated and suspended in RPMI 1640 medium (with 10% FBS, 25 mM HEPES). Cells from tumours were isolated using a previously reported [31] method whereby tumours were minced and incubated with digestion buffer (RPMI 1640 medium, 10% FBS, 25 mM HEPES, 300 Unit Type2 collagenase [Worthington Biochemical, Lakewood, NJ, USA]) in a shaker for 1 h at 37 °C. Dispersed cells were filtered through a 70-μm cell strainer to eliminate clumps and debris. After centrifugation for 3 min (500 *g*), cells from the spleen and tumour were resuspended in red blood cell lysis buffer (G-Biosciences, Louis, MO, USA) and incubated at room temperature for 1 min to remove erythrocytes. Cells were filtered again through a 40 μm cell strainer. Finally, the cells were re-pelleted and re-suspended in RPMI 1640 medium.

### Statistical analysis

The mice were separated to experimental and control group at random. Statistical analysis was performed with SPSS software ver. 25 (IBM, Armonk, NY, USA) using the unpaired two-tailed t-test or Bonferroni’s multiple comparison test. Kaplan-Meier curves were created by the same software and log rank analysis was used for comparison between groups. *P* values less than 0.05 was considered statistically significant.

## Results

### IFNγ increases PD-L1 expression in osteosarcoma cell lines

**Fig. 1:**
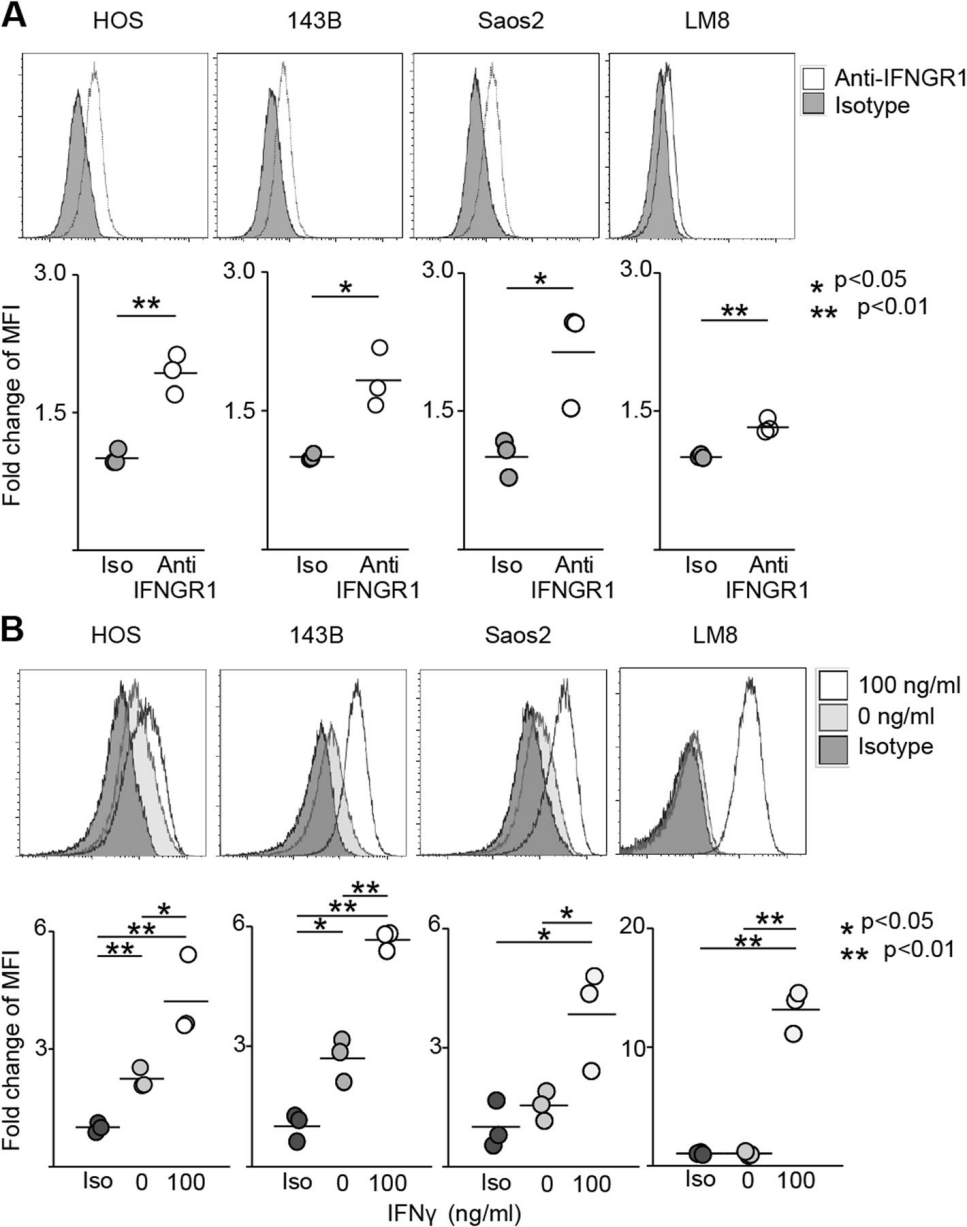
IFNγ increases PD-L1 expression in osteosarcoma cell lines. Surface markers of human and murine osteosarcoma cell line were evaluated by flow cytometry. The upper row shows representative specimen. In the lower row, each specimen is plotted (*n* = 3), and the average value is indicated by a horizontal bar. **a** Expression of IFNGR1 in each cell line. **b** Expression of PD-L1 in each cell line. Iso-type control, anti-PD-L1 staining with/without IFNγ were evaluated

Osteosarcoma cell lines were used to verify the mechanism of PD-L1 expression in osteosarcoma. Analysis of human (HOS, SaOS-2, 143B) and mouse (LM8) osteosarcoma cell lines by flow cytometry revealed that IFNGR1 was expressed in all cell lines (HOS; 1.92, *p* = 0.0022, 143B; 1.83, *p* = 0.011, SaOS-2; 2.14, *p* = 0.027, and LM8; 1.3, *p* = 0.0017. Figure 1a). When IFNγ was applied, PD-L1 expression increased in all cell lines (HOS; 0 group 2.24, 100 group 4.23, *p* value: iso vs 0 = 0.0019, iso vs 100 = 0.0056, 0 vs 100 = 0.031. 143B; 0 group 2.70, 100 group 5.65, *p* value: iso vs 0 = 0.010, iso vs 100 < 0.0001, 0 vs 100 = 0.0010. SaOS-2; 0 group 1.54, 100 group 3.85, *p* value: iso vs 0 = 0.25, iso vs 100 = 0.025, 0 vs 100 = 0.039. LM8; 0 group 0.99, 100 group 13.19, *p* value: iso vs 0 = 0.93, iso vs 100 = 0.00034, 0 vs 100 = 0.00034. Figure 1b). In SaOS-2 and LM8, no significant expression of PD-L1 was observed unless stimulated by IFNγ; in contrast, PD-L1 was expressed in HOS and 143B without IFNγ stimulation.

### Anti-PD-1 antibody improves survival curve in vivo

**Fig. 2:**
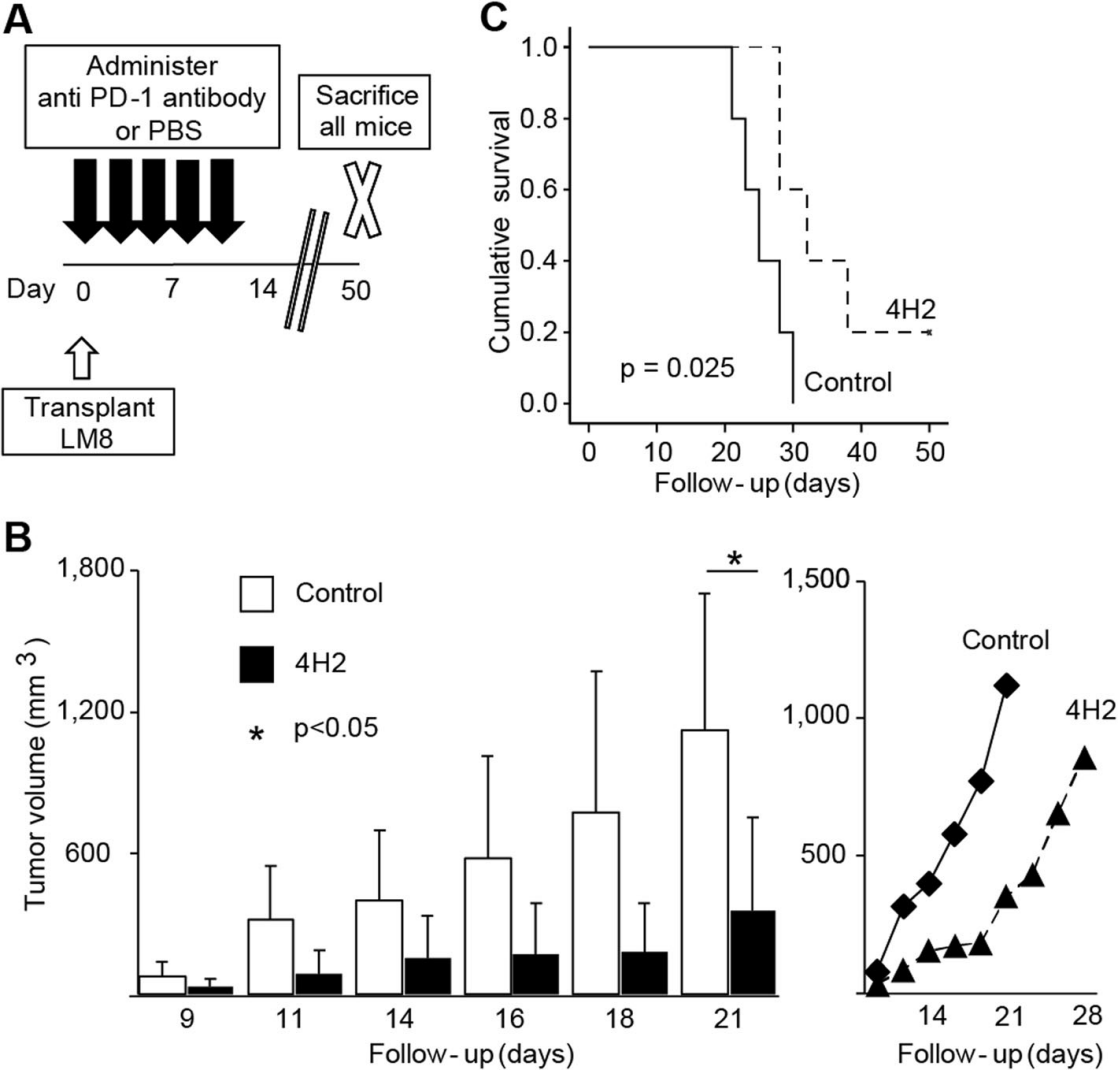
Anti-PD-1 antibody improves survival curve in vivo*.* The LM8 murine osteosarcoma cell line was subcutaneously transplanted to the dorsal region of the C3H mouse, and 4H2 was administered (*n* = 5). Error bars indicate S.D. **a** Schema of experimental overview. **b** Changes in tumour volume with time to first death. The right line graph plots the mean value of the tumour volume. **c** Evaluation of overall survival rate by Kaplan-Meier curve. *P* value of log rank method was 0.025

Anti-PD-1 antibody (4H2) was administered to subcutaneously implanted models of murine osteosarcoma cell line LM8 and evaluated in vivo. First, anti-PD-1 antibody was administered three times per week for a total of five times and compared to the control (Fig. 2a). An increase in tumour volume was suppressed in the 4H2 group, and a significant difference was found at 3 weeks after transplantation (Day 9; control 79.6 ± 59.6 mm^3^, 4H2 33.8 ± 30.5 mm^3^, *p* = 0.16. Day 11; control 317.7 ± 228.9 mm^3^, 4H2 86.0 ± 102.2 mm^3^, *p* = 0.073. Day 14; control 400.2 ± 298.1 mm^3^, 4H2 153.6 ± 177.8 mm^3^, *p* = 0.15. Day 16; control 578.4 ± 435.7 mm^3^, 4H2 172.6 ± 212.9 mm^3^, *p* = 0.098. Day 18; control 771.1 ± 600.9 mm^3^, 4H2 183.3 ± 201.9 mm^3^, *p* = 0.072. Day 21; control 1122.1 ± 579.6 mm^3^, 4H2 352.8 ± 399.9 mm^3^, *p* = 0.040. Figure 2b). The tumour diameter rapidly increased in the 4H2 administration group after day 18 (Fig. 2b, right). Survival curves were significantly improved (*p* = 0.025, Fig. 2c), and mean survival significantly increased from 25.4 ± 1.6 days in the control group to 35.2 ± 3.7 days in the 4H2 group (*p* = 0.047, Fig. 2d).

### Anti-PD-1 antibody changes the tumour microenvironment

**Fig. 3:**
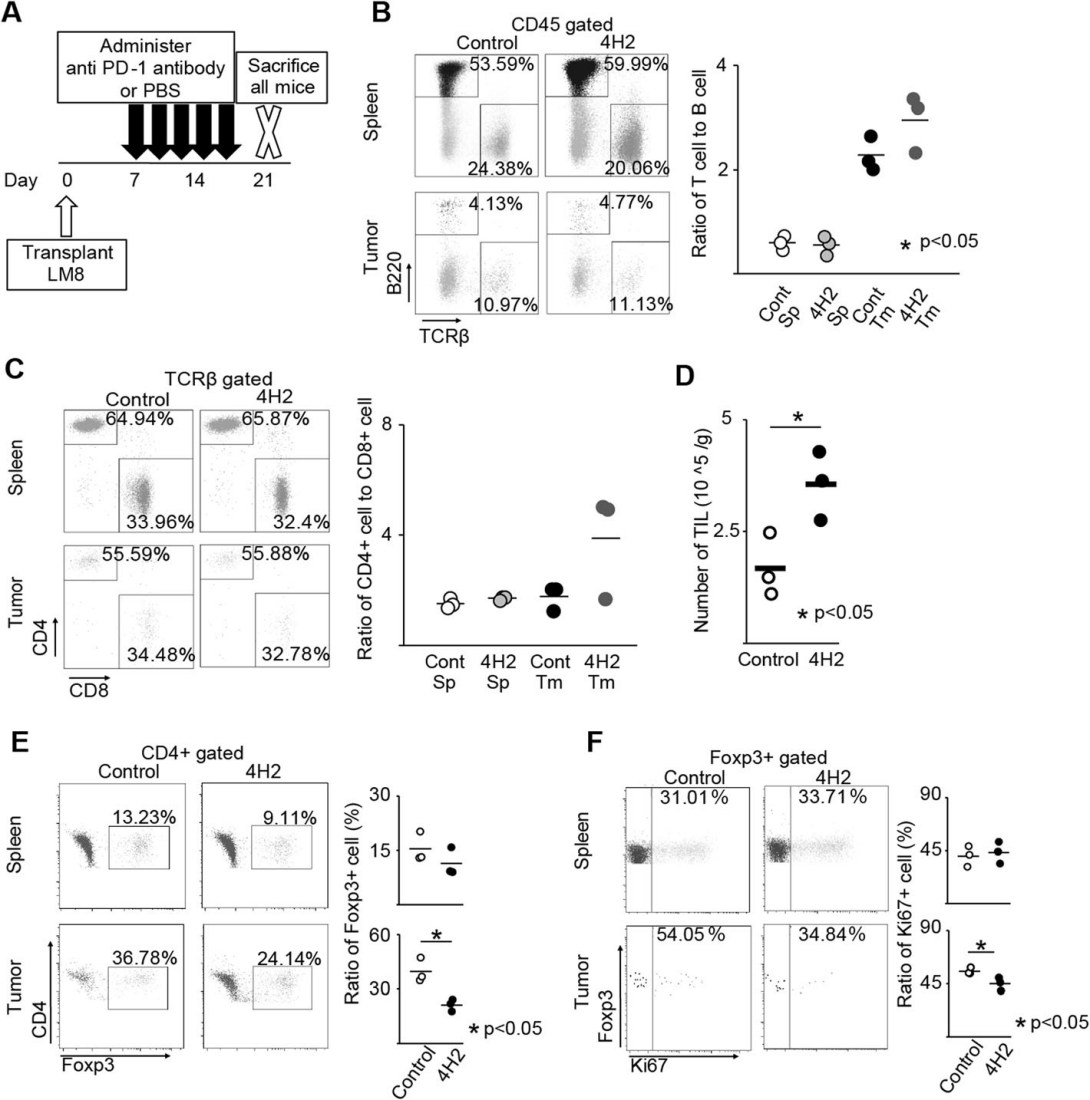
Anti-PD-1 antibody changes the tumour microenvironment. The proportion of spleen and tumour immune cells were evaluated (*n* = 3). The representative specimen is shown, left. Each specimen is plotted, and the average value is indicated by a horizontal bar, right. **a** Schema of experimental overview. **b** Ratio of T cell to B cell. **c** Ratio of CD4 + cell to CD8 + cell in TCRβ + cells. **d** Number of TILs recovered from the tumour per unit weight. **e** Percentage of Foxp3 + cells in CD4 + cells. **f** Percentage of Ki-67 + cells in Foxp3 + cells

To investigate changes in the tumour microenvironment following the administration of anti-PD-1 antibody, all mice were euthanized 2 weeks after the initial administration of anti-PD-1 antibody, spleen and tumour were collected, and cells were isolated (Fig. 3a). Expression of surface and intracellular antigens were evaluated by flow cytometry, and the proportion of immune cells in the spleen and tumour was examined. In addition, focusing on the expression of PD-1 molecule on the surface of Treg that suppresses T cells, we also examined the change in the proportion of Treg in CD4 + cells after the administration of anti-PD-1 antibody.

In the spleen, the administration of anti-PD-1 antibody showed no changes in the ratio of T cell to B cell (Control; 0.53 ± 0.16, 4H2; 0.54 ± 0.19. *p* = 0.73, Fig. 3b), ratio of CD4 + T cell to CD8 + T cell (Control; 1.6 ± 0.29, 4H2; 1.7 ± 0.07. *p* = 0.15, Fig. 3c), and percentage of Foxp3 + Treg in CD4 + cells (Control; 16.5 ± 4.0%; 4H2; 11.5 ± 3.9%. p = 0.15, Fig. 3e). The number of tumour-infiltrating lymphocytes increased per tumour weight after anti-PD-1 antibody administration (Control; 1.7 ± 0.7 × 10^5^ /g, 4H2; 3.6 ± 0.8 × 10^5^ /g. *p* = 0.047, Fig. 3d). Within the tumour, the ratio of T cells in CD45 + cells were higher compared to the spleen (Fig. 3b, right). However, the administration of anti-PD-1 antibody showed no changes in the ratio of B cell to T cell within the tumour (Control; 2.2 ± 0.29, 4H2; 3.0 ± 0.55. *p* = 0.14, Fig. 3b) and the ratio of CD4 + T cell and CD8 + T cell (Control; 1.8 ± 0.37, 4H2; 3.9 ± 1.9. *p* = 0.13, Fig. 3c). On the other hand, the percentage of Foxp3 + Treg in CD4 + T cells within the tumour was significantly decreased with the administration of anti-PD-1 antibody (Control; 39.5 ± 6.7%, 4H2; 21.2 ± 3.2%. *p* = 0.013Fig. 3e). Moreover, Ki67, which is highly expressed in effector Treg that exhibits strong immunosuppressive activity, decreased in the group treated with anti-PD-1 antibody (Control; 55.5 ± 2.9%, 4H2; 45.0 ± 5.7%. *p* = 0.046, Fig. 3f).

### Long-term administration enhances the effect of anti-PD-1 antibody

We found that the long-term administration of anti-PD-1 antibody suppressed tumour volume and prolonged overall survival. Even in the group administered with 4H2 for 2 weeks, the speed of tumour growth increased at approximately 1 week after final administration (Fig. 2b, right). We investigated whether long-term anti-tumour effect can be obtained by continuously administering 4H2 over an extended period of time.

**Fig. 4:**
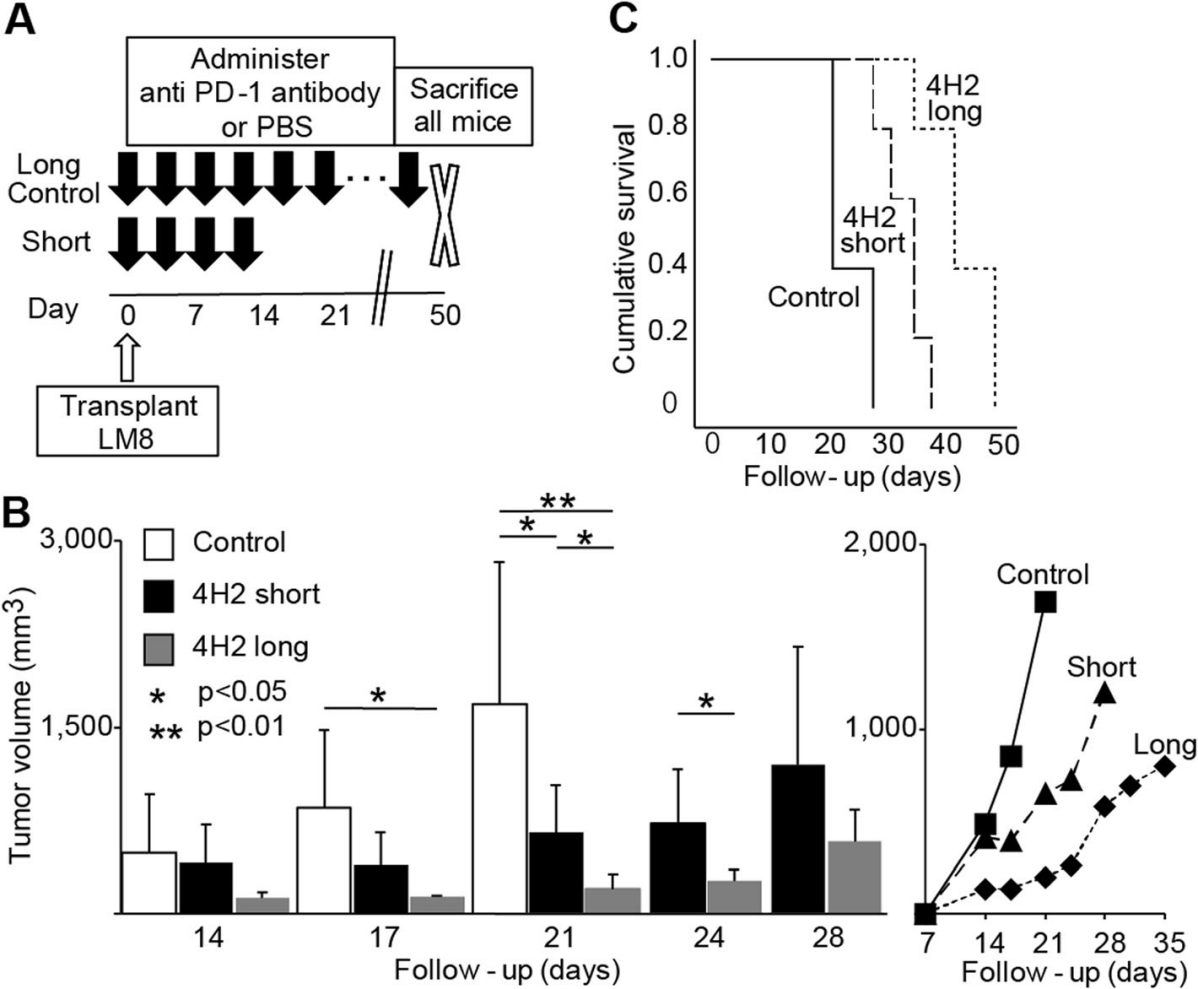
Long-term administration enhances the effect of anti-PD-1 antibody. The control group, the short-term group that was limited to four doses and the long-term group that continued administration until natural death or euthanasia criteria were compared (*n* = 5). Error bars indicate S.D. **a** Schema of experimental overview. **b** Changes in tumour volume with time until first death. The right line graph plots the mean value of tumour volume. **c** Evaluation of overall survival rate by Kaplan-Meier curve. *P* value of log rank method was 0.0002

For the long-term administration group, 4H2 was administered twice a week from day 0 until death or having reached our euthanasia criteria. The results were compared with a control group in addition to a short-term administration group that received only four administrations in total (Fig. 4a). In both the long-term and short-term groups, the increase in tumour volume was suppressed compared to the control group (Day 14; control 487.3 ± 478.6 mm^3^, short-term 412.8 ± 302.8 mm^3^, long-term 132.3 ± 41.2 mm^3^, *p* value: control vs short-term = 0.72, control vs long-term = 0.093, short-term vs long-term = 0.074. Day 17; control 904.8 ± 549.8 mm^3^, short-term 392.9 ± 260.9 mm^3^, long-term 132.8 ± 16.3 mm^3^. *p* value: control vs short-term = 0.097, control vs long-term = 0.014, short-term vs long-term = 0.057. Day 21; control 1844.1 ± 1041.5 mm^3^, short-term 649.7 ± 384.5 mm^3^, long-term 198.3 ± 124.0 mm^3^. *p* value: control vs short-term = 0.043, control vs long-term = 0.0080, short-term vs long-term = 0.037. Day 24; short-term 726.5 ± 430.8 mm^3^, long-term 260.1 ± 92.3 mm^3^. *p* = 0.045. Day 28; short-term 1200.4 ± 952.8 mm^3^, long-term 586.1 ± 253.2 mm^3^. *p* = 0.20. Day 31; long-term 694.4 ± 231.2 mm^3^. Day 35; long-term 800.5 ± 202.9 mm^3^. Figure 4b), the survival curve was significantly improved (*p* = 0.0002. Figure 4c), and the mean days of survival were significantly extended (control; 23.8 ± 3.8 days, short-term; 33.4 ± 3.9 days, long-term; 43.4 ± 5.9 days. *p* value: control vs short-term = 0.0044, control-term vs long-term = 0.00024, short-term vs long-term = 0.013. Figure 4d). Moreover, in a comparison between the long-term and short-term administration groups, the tumour volume in the long-term administration group suppressed further as the survival curve and mean survival period also increased further (Fig. 4b).

## Discussion

In this study, we selected osteosarcoma as a tumour model due to its susceptibility to immunotherapy. First, the pattern of expression for PD-L1 in osteosarcoma cells was confirmed. Next, we verified the effectiveness of anti-PD-1 antibody on osteosarcoma in animal experiments and clarified the mechanism of action of anti-PD-1 antibody by evaluating the tumour microenvironment. The above results demonstrate that osteosarcoma is suitable as a model for evaluating the action of anti-PD-1 antibody on tumour.

Two types of PD-L1 expression in the tumour are known: an innate mechanism induced by genetic mutation and an adaptive mechanism induced by the stimulation of inflammatory cytokine, the latter of which is primarily caused by IFNγ [1]. Constitutive PD-L1 expression, known as innate immune resistance, is created by the deletion or silencing of PTEN found in glioblastoma [32] or by a signal transducer and activator of transcription 3 (STAT 3) signalling found in lymphoma and occasionally in lung cancer [33]. PD-L1 expression by immune response, known as adaptive immune resistance, mainly causes IFNγ stimulation to resolve inflammation in normal tissue at an appropriate time and to avoid excessive tissue damage. Though it is still not clear how much concentration of IFN-γ is appropriate to reproduce tumour microenvironment in vitro sturdy, there are some studies about relationship between tumour and IFN-γ. For example, in ovarian cancer and angiosarcoma, IFNγ induces PD-L1 expression and is known to be involved in immune tolerance [4, 5]. In this study, we found that IFNγ enhances the expression of PD-L1 in osteosarcoma and the result is compatible with other reports. It is known that IFNγ is produced by inflammatory cells such as T cells and NK cells, and this in vitro result shows the possibility that adaptive PD-L1 expression is occurring in response to inflammation of osteosarcoma. In addition, the existence of a negative feedback loop has been reported, whereby PD-L1 is expressed on the tumour surface by T cell activation and IFNγ production, and T cell activity is suppressed via PD-L1 [1]. In osteosarcoma, T-cells produce IFNγ that express PD-L1, and immune tolerance may be caused by a negative feedback loop through the PD-L1. In other words, the inhibition of PD-1/PD-L1 interaction with anti-PD-1 antibody may potentially cause T cells to produce an anti-tumour effect without undergoing negative feedback.

Furthermore, we confirmed the inhibition of tumour growth by anti-PD-1 antibody and the prolonged overall survival for subcutaneously implanted mouse models of osteosarcoma in vivo. These results demonstrate that osteosarcoma is a suitable model for evaluating the effect of anti-PD-1 antibody on tumours.

Many studies have been conducted on changes in the tumour microenvironment during the use of immune checkpoint inhibitors in various carcinomas [34–37]. It has been reported that the infiltration of CD8 + T cells into the tumour is increased by the anti-PD-1 antibody [35]. We showed that the number of tumours infiltrating lymphocytes per unit weight increases in osteosarcoma by the administration of anti-PD-1 antibody, and this result is consistent with previous reports. On the other hand, the relationship between anti-PD-1 antibody and CD4+ is still unclear. Recently, Zuazo et al. reported anti-PD-1 antibody increase CD4+ cells and some reports also showed increasing of CD4+ cells in their data [19, 38]. In our data, though the mechanism is not disclosed, CD4+ cells in tumour tended to increase.

It is widely known that Treg suppresses immunity against tumours in the tumour microenvironment [39]. Treg is characterized by Foxp3 +, and Miyara et al. have described a classification of Foxp3 + T cells and reported that there are many effector Treg fractions in tumour tissue [14]. Effector Treg is considered a fraction with strong immunosuppressive function amongst Foxp3 + T cells and induces high expressions of PD-1 and Ki-67 [15]. Other fractions of Foxp3 + T cells have a weak suppressive function, and expressions of PD-1 and Ki-67 are not observed. The results of this study show that by using anti-PD-1 antibody, the proportion of Foxp3 + T cells in CD4 + cells and Ki-67 + cells in Foxp3 + T cells decreases. This suggests that the administration of anti-PD-1 antibody eliminates effector Treg expressing PD-1 among Foxp3 + T cells. Like PD-1, CTLA-4 is a molecule that expresses a high level of effector Treg. Reduction of Treg by administration of anti-CTLA-4 antibody is well known [12, 13, 37, 40, 41], and the cause is reported to be due to the antibody-dependent cell cytotoxicity (ADCC) of the anti-CTLA-4 antibody [11, 41]. ADCC is the activation of macrophages and NK cells that recognize the Fc region of the antibody to migrate and kill the cells when the antibody binds to cells. Because PD-1 molecule is expressed on Treg surface like that of CTLA-4 [24] and ADCC is similarly caused by binding to anti-PD-1 antibody, the administration of anti-PD-1 antibody to decrease Treg has been hypothesized for a long time [42], but have not yet to be proven. Recently some reports mentioned about the relationship between anti-PD-1 antibody and Treg, but it is still controversial. Kamada et al. reported the tendency of decrease of Treg after anti-PD-1 therapy for gastric cancer patients. Asano et al. reported the importance of PD-1 on the proliferation of Treg by using anti-PD-1 antibody. Zhang et al. reported PD-1-dificient Treg showed increased their suppressive activity in Foxp3 decreased mouse and concluded to affect the expression of both PD-1 and FoxP3 can disrupt the T-cell homeostasis [17]. Here, we showed for the first time that Treg decreases by administration of anti-PD-1 antibody in osteosarcoma. Moreover, as there are no other reports that clearly describe the decrease in Treg as a result of administrating anti-PD-1 antibody in other types of tumours, this is the first discovery of its kind in the world.

In this study, we showed that the survival time of subcutaneously transplanted mouse models of osteosarcoma was prolonged by the administration of anti-PD-1 antibody. Furthermore, the survival time is likely to extend further with a long-term administration of anti-PD-1 antibody. As expected, long-term administration of anti-PD-1 antibody was found to prolong survival time compared to short-term administration in this study.

We revealed for the first time that anti-PD-1 antibody suppresses the infiltration of Treg into tumour and exerts an antitumour effect. Although osteosarcoma was used as a tumour model in this study, future studies should investigate these results further in the context of other tumours. If the mechanism of action in tumour suppression of anti-PD-1 antibody is established in many types of cancer, it will enable us to provide an explain regarding the higher response rate of anti-PD-1 antibody compared to anti-PD-L1 antibody in clinical practice [10]. In terms of clinical application, these findings might also change the effect prediction of anti-PD-1 antibody based on the expression of PD-L1 on the tumour surface because of its antitumour ability via decreasing Treg.

We believe that this study not only affects the use of anti-PD-1 antibody in clinical practice, but also serves to facilitate the development of new treatments for tumours with a focus on the relationship between anti-PD-1 antibody and Treg.

It is limitation of this study that the most results are from mouse transplantation model of osteosarcoma. Because it is difficult to be proven this model behaves the same as the human disease, further studies like using humanized mice or collecting the data from clinical specimen of osteosarcoma used anti-PD-1 antibody are needed.

## Conclusions

The suppression of Treg by the administration of anti-PD-1 antibody is a new finding and may offer new insights into the mechanism of action of the anti-PD-1 antibody. The results of this study could be a useful asset in designing a combination therapy of anti-PD-1 antibody with other drugs. Moreover, because an antitumour effect can be anticipated through the suppression of Treg, clinical importance can also be placed in the possible therapeutic effects for tumours with no expression of PD-L1.

## Supplementary information

**Additional file 1: Table S1.** List of antibodies.

## Data Availability

Data presented in this manuscript are available from the corresponding author upon reasonable request.

## Abbreviations

ADCC: Antibody-dependent cell cytotoxicity
APC: Antigen-presenting cell
CTLA-4: Cytotoxic T-lymphocyte antigen 4
DMEM: Dulbecco’s Modified Eagle Medium
FBS: Fetal bovine serum
IFN: Interferon
LAG-3: Lymphocyte activation gene 3
PD-1: Programmed cell death 1
PD-L1: Programmed cell death ligand 1
STAT3: Signal transducer and activator of transcription 3
