# Supplementary figures and images for: Anti-PD-1 antibody decreases tumour-infiltrating regulatory T cells
Source: BMC Cancer. 2020 Jan 8;20:25. doi: 10.1186/s12885-019-6499-y (PMC6950856; doi:10.1186/s12885-019-6499-y)

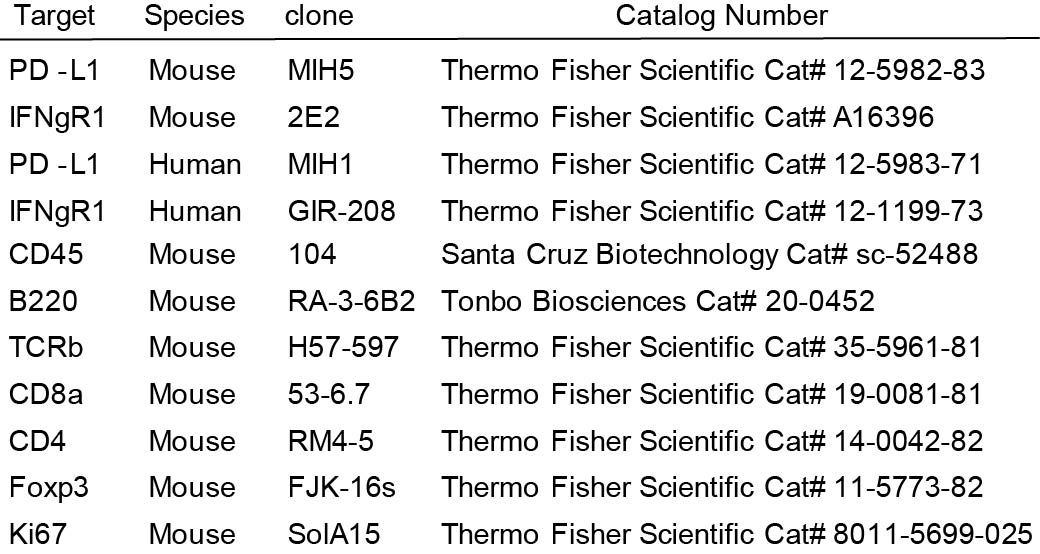

Supplement: Supplementary file 1 — Additional file 1: Table S1. List of antibodies. [file 12885_2019_6499_MOESM1_ESM.jpg]
